# Postprandial Fatty Acid Profile, but Not Cardiometabolic Risk Markers, Is Modulated by Dairy Fat Manipulation in Adults with Moderate Cardiovascular Disease Risk: The Randomized Controlled REplacement of SaturatEd fat in dairy on Total cholesterol (RESET) Study

**DOI:** 10.1093/jn/nxab050

**Published:** 2021-03-23

**Authors:** Oonagh Markey, Dafni Vasilopoulou, Kirsty E Kliem, Colette C Fagan, Alistair S Grandison, Rachel Sutton, David J Humphries, Susan Todd, Kim G Jackson, David I Givens, Julie A Lovegrove

**Affiliations:** Hugh Sinclair Unit of Human Nutrition and Institute for Cardiovascular and Metabolic Research, University of Reading, Reading, United Kingdom; Hugh Sinclair Unit of Human Nutrition and Institute for Cardiovascular and Metabolic Research, University of Reading, Reading, United Kingdom; Animal, Dairy, and Food Chain Sciences, University of Reading, Reading, United Kingdom; Institute for Food, Nutrition, and Health, University of Reading, Reading, United Kingdom; Hugh Sinclair Unit of Human Nutrition and Institute for Cardiovascular and Metabolic Research, University of Reading, Reading, United Kingdom; Institute for Food, Nutrition, and Health, University of Reading, Reading, United Kingdom; Hugh Sinclair Unit of Human Nutrition and Institute for Cardiovascular and Metabolic Research, University of Reading, Reading, United Kingdom; Hugh Sinclair Unit of Human Nutrition and Institute for Cardiovascular and Metabolic Research, University of Reading, Reading, United Kingdom; Animal, Dairy, and Food Chain Sciences, University of Reading, Reading, United Kingdom; Institute for Food, Nutrition, and Health, University of Reading, Reading, United Kingdom; Department of Mathematics and Statistics, University of Reading, Reading, United Kingdom; Hugh Sinclair Unit of Human Nutrition and Institute for Cardiovascular and Metabolic Research, University of Reading, Reading, United Kingdom; Institute for Food, Nutrition, and Health, University of Reading, Reading, United Kingdom; Institute for Food, Nutrition, and Health, University of Reading, Reading, United Kingdom; Hugh Sinclair Unit of Human Nutrition and Institute for Cardiovascular and Metabolic Research, University of Reading, Reading, United Kingdom; Institute for Food, Nutrition, and Health, University of Reading, Reading, United Kingdom

**Keywords:** endothelial function, monounsaturated fatty acids, postprandial lipemia, saturated fatty acids, sequential test meal protocol

## Abstract

**Background:**

Chronic consumption of dairy products with an SFA-reduced, MUFA-enriched content was shown to impact favorably on brachial artery flow-mediated dilatation (FMD). However, their acute effect on postprandial cardiometabolic risk biomarkers requires investigation.

**Objective:**

The effects of sequential high-fat mixed meals rich in fatty acid (FA)–modified or conventional (control) dairy products on postprandial FMD (primary outcome) and systemic cardiometabolic biomarkers in adults with moderate cardiovascular risk (≥50% above the population mean) were compared.

**Methods:**

In a randomized crossover trial, 52 participants [mean ± SEM age: 53 ± 2 y; BMI (kg/m^2^) 25.9 ± 0.5] consumed a high-dairy-fat breakfast (0 min; ∼50 g total fat: modified: 25 g SFAs, 20 g MUFAs; control: 32 g SFAs, 12 g MUFAs) and lunch (330 min; ∼30 g total fat; modified: 15 g SFAs, 12 g MUFAs; control: 19 g SFAs, 7 g MUFAs). Blood samples were obtained before and until 480 min after breakfast, with FMD assessed at 0, 180, 300, and 420 min. Data were analyzed by linear mixed models.

**Results:**

Postprandial changes in cardiometabolic biomarkers were comparable between the different dairy meals, with the exception of a tendency for a 4% higher AUC for the %FMD response following the modified-dairy-fat meals (*P* = 0.075). Plasma total lipid FA analysis revealed that incremental AUC responses were 53% lower for total SFAs, 214% and 258% higher for total *cis*-MUFAs (predominantly *cis*-9 18:1), and *trans*-18:1, respectively, following the modified relative to the control dairy meals (all *P* < 0.0001).

**Conclusions:**

In adults at moderate cardiovascular risk, acute consumption of sequential high-fat meals containing FA-modified dairy products had little impact on postprandial endothelial function or systemic cardiometabolic biomarkers, but a differential effect on the plasma total lipid FA profile, relative to conventional dairy fat meals.

This trial was registered at clinicaltrials.gov as NCT02089035.

## Introduction

The replacement of dietary SFAs with unsaturated fatty acids (FAs) is advised as a key public health strategy for cardiovascular disease (CVD) risk reduction (1, 2). Milk and dairy products are a major source of SFAs in the diet (3). However, it is known that the food matrix can influence the nutritional properties, nutrient absorption, and associated health effects of an SFA-rich food (e.g., cheese) (4). Indeed, evidence from prospective studies and meta-analyses indicates that full-fat dairy products are not linked to increased CVD risk, except for butter fat (4). As dairy products play a substantial role in meeting essential nutrient requirements, limiting their consumption to reduce SFA intake may inadvertently affect public health (5–7).

Exaggerated postprandial triacylglycerol (TG) is an independent CVD risk factor, with high-fat meals known to lead to a transient increase in TG-rich lipoproteins during the postprandial phase (8, 9). A meta-analysis of postprandial lipemic responses to single high-fat meals highlighted that, over an 8-h period, there was a benefit or a tendency towards a beneficial effect of replacing SFAs (mainly butter) by either PUFA- or MUFA-rich oils, respectively (10). Additionally, the type of dairy product incorporated into fat- and FA-matched meals has been shown to differently affect the postprandial TG response, with sour cream inducing a larger TG incremental AUC (iAUC) over 6 h compared with whipped cream, butter, and cheese (11). However, little is known about how manipulating the FA composition of high-dairy-fat load affects postprandial lipemia, particularly using a sequential 2-meal protocol that contains a combination of commonly consumed dairy products and, thus, more closely reflects real-life eating patterns.

Postprandial TG may trigger a cascade of proinflammatory events that lead to endothelial dysfunction, an early event in the pathogenesis of atherosclerosis (12, 13). Although single high-fat meals (36–80 g total fat) are known to impair postprandial endothelial function, including flow-mediated dilatation (FMD) (14), evidence regarding the comparative effects of test meals rich in unsaturated FAs with SFAs or different dairy products on postprandial FMD or biomarkers of endothelial function is inconclusive (14, 15).

In a proof-of-concept study, we recently reported that 12-wk intake of SFA-reduced, MUFA-enriched milk, cheese, and butter (∼41 g dairy fat/d) that were FA-modified via a dairy-cow feeding strategy (16), attenuated the rise in fasting LDL-cholesterol concentrations and had a beneficial effect on FMD, relative to conventional dairy product consumption (17). As individuals spend up to 18 h/d in the nonfasting state (9, 18), there is a clear need to gain an understanding of the postprandial cardiometabolic responses to FA-modified dairy products (5). Therefore, we investigated the acute effect of sequential high-fat mixed meals containing FA-modified dairy products on the postprandial %FMD response, systemic cardiometabolic risk biomarkers, and plasma total lipid FA responses in adults with moderate CVD risk. We hypothesized that meals rich in FA-modified milk, cheese, and butter would beneficially impact on the postprandial %FMD response (primary outcome) and other cardiometabolic risk biomarkers compared with matched meals containing dairy foods with an FA profile typical of conventional retail products (control).

## Methods

### Participants

The REplacement of SaturatEd fat in dairy on Total cholesterol (RESET) Study was performed at the Hugh Sinclair Unit of Human Nutrition, University of Reading (Berkshire, UK). Men and women [aged 25–70 y; BMI (kg/m^2^)19–32] at moderate CVD risk were recruited from the Berkshire area between February 2014 and September 2015. To assist with participant recruitment, the study upper age limit was extended from 65 y to 70 y in May 2014. Details of the participant recruitment strategy, inclusion and exclusion criteria, and dietary intervention are presented in detail elsewhere (17, 19). In brief, a modified Framingham CVD risk score identified individuals at moderate risk of developing CVD (≥50% greater CVD risk compared with the population mean) (19). Eligible participants were nonsmokers with mild/moderate hypercholesterolemia (fasting total cholesterol ≥5.2–8.0 mmol/L); not diagnosed with CVD and diabetes (and fasting glucose concentration <7 mmol/L); not taking medication for hyperlipidemia, hypertension, hypercoagulation, or inflammation; not pregnant or lactating; not consuming excessive amounts of alcohol (<14 and 21 units/wk for women and men, respectively); not regularly engaging in vigorous-intensity aerobic activities (>3 times × 20 min/wk); and presenting with normal biochemistry for liver and kidney function (19). The study was given a favorable ethical opinion for conduct by the University of Reading's Research Ethics Committee (project 13/43), registered with clinicaltrials.gov (NCT02089035), and carried out in accordance with the Declaration of Helsinki of 1975, as revised in 1983. Participants provided written informed consent before enrollment.

### Study design

This trial was an acute randomized, double-blind, sequential-meal, crossover dietary study nested within the larger RESET proof-of-concept chronic intervention study, which examined the impact of long-term consumption of FA-modified dairy products on novel and traditional markers of CVD risk (17). The chronic intervention study consisted of two 12-wk dietary periods where FA-modified or control dairy products were consumed in a randomized crossover manner, separated by an 8-wk washout period (17). As outlined in **Supplemental Figure 1**, the 2 baseline acute study visits were performed prior to commencing each 12-wk dietary intervention period. With the addition of the 8-wk washout period, the duration between the acute study visits examined in the present trial was 20 wk. None of the results reported here for our subcohort have been previously published. Minimization was used to randomly allocate participants to their first treatment (17, 19). The researchers responsible for conducting and/or analyzing measurements (OM, DV, KEK and RS) and the participants were blinded to treatment allocations.

### Postprandial 2-meal protocol

A sequential 2-meal protocol was used to examine the effect of dairy FA manipulation on postprandial endothelial function and cardiometabolic responses. The details of our high-oleic sunflower oil (HOS) dairy-cow feeding strategy, as well as the production, FA profile, and consumer acceptance of the FA-modified milk and dairy products, are published elsewhere (16, 20).

The energy, macronutrient, and FA compositions of the sequential high-fat mixed meals consumed at breakfast and lunch are presented in **Table 1**. Nutritional analysis (energy and macronutrient content) of modified and control dairy samples was conducted in duplicate by SGS United Kingdom Ltd. (Ealing, London; ISO 17,025 accredited laboratory), as described elsewhere (16, 20). In brief, total fat content was determined by low-resolution proton nuclear magnetic resonance. Protein content was calculated by multiplying the total nitrogen, based on the Dumas method, using a standard nitrogen conversion factor of 6.25 to account for the fraction of nonprotein nitrogen in each sample (21). Carbohydrate content was calculated by difference using the Atwater general system (22). Using a standardized procedure (16), lipids extracted from the modified and control dairy products were analyzed in triplicate for FA composition by GC (Bruker 350; Bruker), equipped with a flame ionization detector (FID) and 100-m fused silica capillary column (CP-SIL 88; Agilent Technologies). The breakfast test meal consisted of a toasted sandwich prepared with white bread (75 g; Kingsmill; Allied Bakeries UK), Cheddar cheese (32.6 g) and butter (modified: 32.6 g; control: 29.4 g), cornflakes (38 g, Kellogg's UK) served with ultra-high temperature (UHT) milk (195 g), and a milkshake prepared with UHT milk (330 g) and strawberry sauce (19 g; Askeys; Silver Spoon Company UK). The lunch test meal consisted of a toasted sandwich prepared with white bread (60 g) with cheddar cheese (15 g) and butter (modified: 19.8 g; control: 18.6 g) and a milkshake prepared with UHT milk (modified: 352 g; control: 350 g) and strawberry sauce (27 g).

**TABLE 1:** Nutritional composition of the sequential high-fat mixed test breakfast (0 min) and lunch meals (330 min) that incorporated the fatty acid–modified or conventional (control) dairy products^1^

	Modified			Control		
	Breakfast	Lunch	Total	Breakfast	Lunch	Total
Energy,^2^ MJ	4.3	2.6	6.9	4.1	2.5	6.6
Total fat,^2^ g	50.6	30.6	81.2	49.9	30.3	80.2
SFAs,^3^ g	24.5	14.8	39.3	31.7	19.1	50.8
MUFAs,^3^ g	20.0	12.1	32.1	12.3	7.4	19.7
PUFAs,^3^ g	2.9	1.8	4.7	2.8	1.8	4.6
TFAs,^3^ g	3.9	2.6	6.5	2.2	1.4	3.6
Protein,^2^ g	36.1	20.9	57.0	39.7	19.6	59.3
CHO,^2^ g	105.9	64.6	170.5	101.4	63.3	164.7
Free sugars, g	16.5	22.3	38.8	15.0	21.5	36.5

1 Values are total energy and macronutrient quantities of each test meal according to modified and control diet. CHO, carbohydrate; FA, fatty acid; TFA, *trans* fatty acid.

2 Measurement of energy, total fat, protein, and carbohydrate content of the dairy product samples was conducted in duplicate by SGS UK Ltd. (Ealing, London; ISO 17,025 accredited laboratory).

3 Lipids extracted from the dairy product samples were analyzed in triplicate for FA composition by GC–flame ionization detection using a standardized procedure, as described previously (16).

### Study visits

Participants completed a 4-d weighed food diary to assess habitual dietary intake in the 1-wk run-in period to each study visit, which was analyzed using Dietplan 7 Professional (Forestfield Software Ltd.) (19). Habitual physical activity was assessed using the last 7-d version of the International Physical Activity Questionnaire (IPAQ) long form. For 24 h before each study visit, participants were instructed to avoid alcohol and aerobic exercise and were provided with a low-fat evening ready meal (<1.46 MJ; <7 g total fat) to standardize short-term fat intake. Fasted (12 h) participants arrived at the Clinical Unit after only drinking low-nitrate water overnight (Buxton Mineral Water; Nestlé Waters UK).

Postprandial study visits (480 min) took place in a temperature-controlled clinical room maintained between 22° ± 1°C. Following body-composition assessment, a cannula was inserted into an antecubital vein of the forearm to allow for frequent blood sampling. After a baseline blood sample and a 30-min period of supine rest, endothelial function was assessed by FMD. Subsequently, a second baseline sample was drawn (0 min) before a standardized breakfast test meal was provided and consumed within a period of 20 min. Blood samples were collected at regular intervals (every 30 min until 180 min, and then every 60 min until 300 min). A standardized lunch test meal was provided immediately after the 330-min blood draw and was consumed within 15 min. Blood samples were then collected every 30 min up to 420 min, with the final sample obtained at 480 min after the breakfast meal. FMD was assessed immediately after obtaining blood samples at 180, 300, and 420 min postprandially. Participants remained in the Clinical Unit for the duration of the study visit and were continuously monitored. Low-nitrate water was permitted ad libitum during the study visits.

### Assessment of endothelial function

Three-lead electrocardiogram-gated brachial artery FMD measurements were conducted in a standardized manner using a CX50 CompactXtreme Ultrasound System (Philips HealthCare, UK), as previously reported (17, 23). For each participant, the initial baseline scan image (visit 1) was used as a visual aid to help position the probe on a similar section of the artery for subsequent FMD measurements. Image sequences were assessed in a blinded manner using automated wall-tracking software (Vascular Research Tools 5; Medical Imaging Applications LLC). The %FMD response was calculated at each time point as the relative increase in brachial artery diameter from baseline to maximum dilation, expressed as a percentage.

### Biochemical analyses

Plasma/serum separation was carried out, as previously detailed (17), and samples were subsequently stored at −80°C until analysis. For determination of serum TG, nonesterified fatty acids (NEFAs), insulin, and glucose, blood was drawn at all time points. Samples for serum apoB were collected at 0, 60, 120, 180, 240, 300, 360, 390, 420, and 480 min. At 180, 300, and 420 min (i.e., the designated FMD time points), blood samples were also collected for the assessment of plasma nitrite, nitrate, markers of endothelial activation, total lipid FA responses, as well as whole blood culture for determination of LPS-stimulated cytokine production.

Serum TG, apoB, NEFA, and glucose concentrations were quantified on an ILAB 600 autoanalyzer (Instrumentation Laboratory Ltd.) with the use of colorimetric assay kits (TG and glucose reagents: Instrumentation Laboratory Ltd.; NEFA reagent: Alpha Laboratories Ltd.) and the use of an immunoturbidimetric assay (apoB reagent: Randox Laboratories Ltd.). Serum insulin concentrations were determined by ELISA (Dako UK Ltd.). Plasma nitrite and nitrate concentrations were measured by HPLC (ENO-30; EiCom Corporation) with online reduction of nitrate to nitrite and subsequent post-column derivatization with the Griess reagent (ENO-30 Analyzer; Eicom Corporation) (17). Plasma soluble vascular cell adhesion molecule 1 (sVCAM-1), soluble intercellular adhesion molecule 1 (sICAM-1), E-selectin, and P-selectin concentrations were quantified by the Human Adhesion Molecule Magnetic Luminex Performance Assay 4-Plex kit (R&D Systems Europe Ltd.) with the use of Luminex 200 (Invitrogen) and xPONENT software 3.1 (Luminex). All samples for each participant were analyzed within a single run or kit to reduce interbatch variations. Mean inter-assay CVs were <5% for the ILAB automated assays and <10% for all other analyses.

### LPS-stimulated cytokine analyses

For the determination of whole blood culture LPS-stimulated cytokines, blood samples collected into K_2_-EDTA tubes were not centrifuged and stored at 4°C until processing as previously described (24). Briefly, whole-blood samples were diluted 1:9 with Roswell Park Memorial Institute 1640 medium (Sigma) supplemented with 1% antibiotics, 1% l-glutamine, and 1% nonessential amino acids (Bioscience). Diluted blood samples were cultured in 12-well plates (Greiner Bio-one) with 0.5 μg bacterial LPS/mL (*Escherichia coli* 026:B6; Sigma), at a final concentration of 0.05 μg/mL. Whole blood cultures were subsequently incubated at 37°C for 24 h before centrifugation at 700 × *g* (1000 rpm) for 5 min at room temperature to isolate the supernatant, which was stored at −20°C until analysis. Measurement of the monocyte count of each sample was performed by the Pathology Department at the Royal Berkshire Hospital (Reading, UK). A human cytokine premixed 5-Plex Panel (TNF-α, IL-6, IL-8, IL-1β, IL-10; R&D Systems Europe Ltd.) and a Luminex 200 with xPONENT software 3.1 was used to measure the concentrations of cytokines in the whole blood culture supernatant in a 1:2 dilution. Cytokine production was corrected for the number of monocytes in the whole-blood sample (micrograms × 10^3^ monocytes).

### Total lipid FA analyses

To assess changes in plasma FA status in response to our sequential 2-meal fat challenge, we measured the plasma total lipid FA pool; this pool is representative of immediate FA intake and gives an indication of all FA-containing plasma lipid fractions, including cholesteryl esters, NEFAs, phospholipids, and TG (25). Total lipid in 0.5 mL K_3_-EDTA plasma was extracted using 5.0 mL chloroform-methanol [2:1, vol:vol; Burdge et al. (24)]. After shaking for 15 min, the aqueous and solvent layers were separated using centrifugation (1125 × *g* for 10 min at 4°C), and the solvent layer dried under nitrogen at 40°C. Dried lipid was redissolved in 0.5 mL toluene and methylated by the addition of 1.0 mL 2% H_2_SO_4_ in methanol, and incubated for 2 h at 50°C. After neutralization, FAMEs were dissolved in hexane and centrifuged (1125 × *g* for 10 min at 4°C). Resulting FAMEs were separated on a 100-m fused silica capillary column (CP-SIL 88; Agilent Technologies) using GC (Bruker 350; Bruker) with FID (26). Plasma FAMEs were identified based on retention time comparisons with an authentic standard (GLC #463; Nu-Chek-Prep, Inc.) and cross-referenced with previously published chromatograms (27). Carbon deficiency in the FID response for FAMEs containing 4- to 10-carbon atoms was accounted for using a combined correction factor, which also converted FAMEs to FAs (28).

### Power calculation and statistical analyses

An a priori power calculation was performed for the nested study's primary outcome: change in the postprandial %FMD response. At 80% power and 5% significance, the minimum number of participants required to complete both arms of the study in order to detect a 1.4% difference in the postprandial %FMD response between dairy fat treatments with an SD of 2.3% was calculated as *n* = 45 (29). To allow for a 15% drop-out rate, 52 participants were recruited into the study and randomly assigned. A *P* value <0.05 was considered significant for the primary outcome measure (postprandial %FMD response; *n* = 45). No formal sample-size calculations were performed for secondary outcomes variables, which included lipid metabolism [TG, apoB, NEFA (*n* = 43–49)], glucose and insulin responses (*n* = 46), circulating biomarkers of endothelial activation and inflammation [nitrite and nitrate (*n* = 49); sVCAM-1, sICAM-1, E-selectin, and P-selectin (*n* = 50); whole blood culture LPS-stimulated cytokines (TNF-α, IL-6, IL-1β, IL-8, IL-10) ( *n* = 43–48)], and plasma total lipid FA responses (*n* = 47–48). The FAs deemed relevant to our dietary intervention (from 65 identified FAs) were selected for statistical analysis a priori (19). *P* ≤ 0.01 was chosen a priori when assessing significance for secondary outcome variables to acknowledge the issue of multiplicity (17). Data presented in the text, tables, and figures represent the unadjusted means ± SEMs, unless otherwise stated. We chose to follow a per-protocol analysis approach a priori, as this was a proof-of-concept study rather than a confirmatory trial (17). This approach was taken, rather than intention-to-treat, as the trial was designed to test efficacy (biological effects) rather than effectiveness (30).

Summary measures of postprandial responses included the following: AUC (calculated using the trapezoidal rule) and iAUC (calculated by subtracting the fasting value from the AUC). Inclusion of the iAUC summary measure provided an indication of the change in the postprandial response to the sequential meal ingestion (31). The time interval for AUC and iAUC was 420 min for %FMD, plasma nitrite, nitrate, cell adhesion molecules, whole blood culture LPS-stimulated cytokines, and total lipid FA responses and 480 min for serum TG, apoB, glucose, and insulin responses. Due to the shape of the postprandial NEFA time-course profile, AUC and iAUC for this outcome were calculated from 120 min (approximate time of NEFA minimum concentration) to 480 min postprandially (i.e., 360-min interval) (32).

For postprandial variables with 13 (TG, NEFA, glucose, and insulin) or 10 (apoB) time points, maximum (peak) concentration reached after the test meal (Cmax) and time to reach maximum concentration (Tmax) were also calculated. Additional outcome measures for NEFAs included minimum concentration reached after the first test meal (Cmin), time to reach minimum concentration (Tmin), and percentage of NEFA suppression from fasting (32). A participant's study visit data were excluded from biochemical analyses if ≥40% of data or 3 consecutive blood time points were missing. Since ingestion of sequential meals leads to biphasic lipid and glycemic control responses (33, 34), AUC and iAUC were also calculated for the time period between breakfast and lunch (0–330 min) and post-lunch (330–480 min) for serum TG, apoB, glucose, and insulin responses (i.e., variables with ≥10 time points). As NEFA AUC and iAUC responses were calculated from 120 to 480 min post-breakfast, it was not considered physiologically relevant to calculate before and after the lunch for this outcome.

Before analysis, suitable checks for normality were conducted on all variables and the ln transformation was used for skewed outcomes. Statistical analyses were performed with the use of SAS 9.4 University edition statistical software (SAS Institute, Inc.). The baseline characteristics prior to the modified and control study visits were assessed by paired *t* tests and chi-square test for continuous and categorical variables, respectively. For all primary and secondary outcomes, linear mixed-model analyses (PROC MIXED) were used to evaluate fasting and treatment effects of meal dairy FA composition on postprandial summary data. Postprandial time-course responses to the meal dairy FA composition were also assessed using linear mixed models. Treatment × time interaction effects were initially included in the model and retained where found to be significant. In the absence of a significant interaction, the interaction term was removed from the model so that the overall treatment effect could be assessed. Fixed (period, time, treatment, gender, age, and BMI) and random (participant) effects were retained in all linear mixed models, regardless of their degrees of significance.

## Results

### Study participation

**FIGURE 1:**
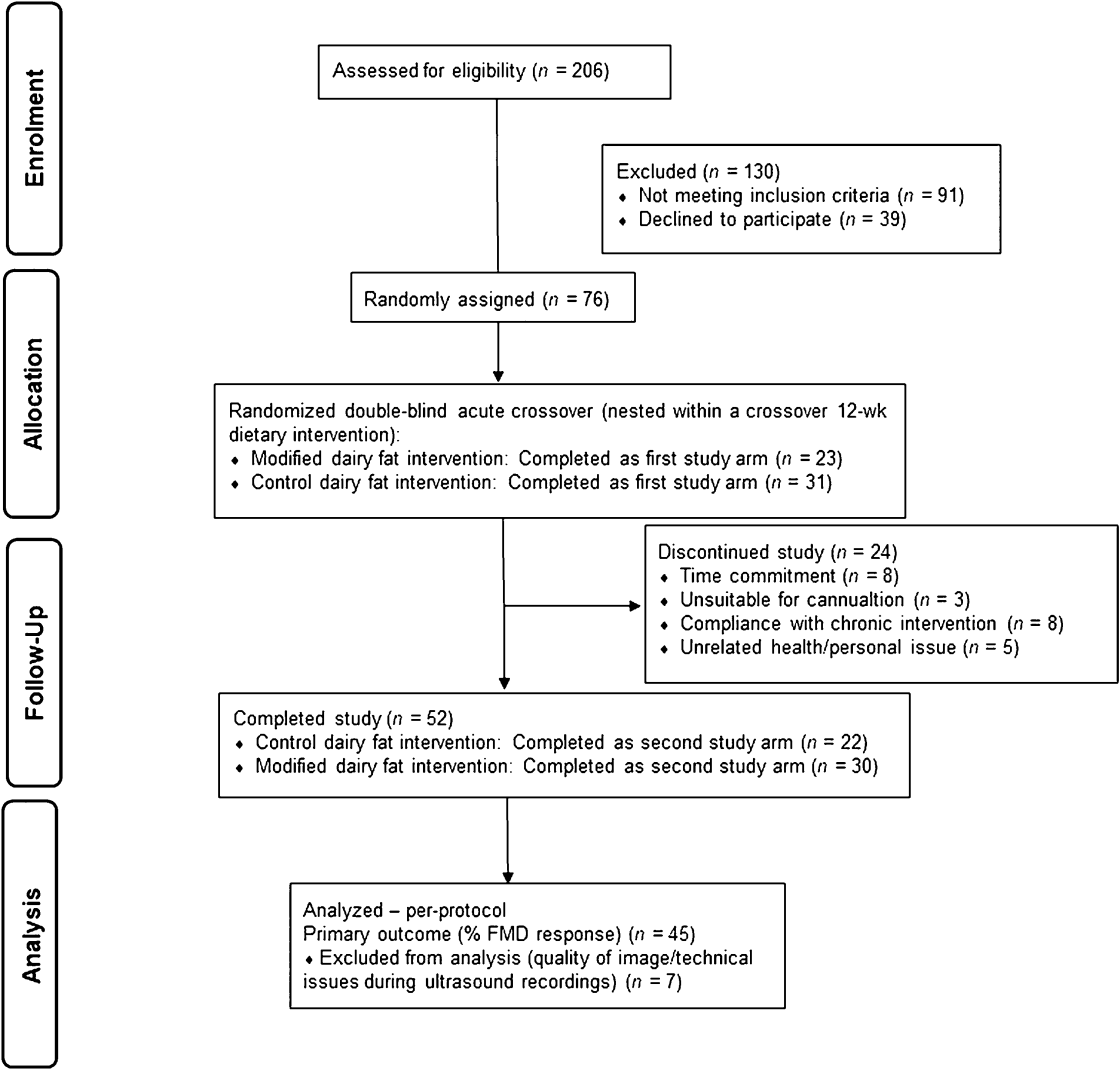
Flow of participants through the study. %FMD, percentage of flow-mediated dilatation response.

A total of 52 participants successfully completed the study (see **Figure 1** for flowchart). There were no large biases in the sex and age of individuals who were randomly assigned to the intervention (*n* = 76) and those who completed the intervention (*n* = 52; data not shown). Baseline characteristics of participants at the beginning of each acute study visit are presented in **Table 2**. The mean CVD risk score of participants at screening, as assessed with the use of a modified Framingham risk score, was 3.0 ± 0.2. As outlined in Table 2, there were no differences in the baseline characteristics or the habitual dietary intake of participants prior to the modified and control study visits (*P* > 0.01). Similarly, physical activity scores (as assessed by IPAQ) did not significantly differ prior to the study visits (data not shown). The sequential test meals were fully consumed and well tolerated by all participants, without side effects.

**TABLE 2:** Baseline characteristics of participants at the beginning of each acute study visit^1^

Characteristics	Modified	Control
Sex, *n* (%)		
Men	31 (59.6)	
Women	21 (40.4)	
Age, y	53 ± 2	
Ethnicity, *n* (%)		
White	46 (88)	
Black	2 (4)	
Chinese/Far Eastern	2 (4)	
Asian	2 (4)	
Body weight, kg	77.6 ± 1.9	77.8 ± 1.9
BMI, kg/m²	25.9 ± 0.5	25.9 ± 0.5
Waist circumference, cm	90.1 ± 1.5	89.2 ± 1.4
Blood pressure, mm Hg		
Systolic	120 ± 2	120 ± 2
Diastolic	69 ± 1	70 ± 1
Fasting serum lipid profile, mmol/L		
TC	5.55 ± 0.14	5.48 ± 0.12
LDL-C	3.46 ± 0.11	3.44 ± 0.10
HDL-C	1.52 ± 0.05	1.52 ± 0.04
Habitual dietary intake^2^		
Energy, MJ/d	8.3 ± 0.3	8.5 ± 0.4
Total fat, %TE	35.9 ± 0.8	36.5 ± 0.9
SFAs, %TE	14.1 ± 0.5	13.9 ± 0.5
MUFAs, %TE	11.8 ± 0.4	11.9 ± 0.4
n–6 PUFAs, %TE	4.0 ± 0.2	4.6 ± 0.2
n–3 PUFAs, %TE	0.7 ± 0.0	0.8 ± 0.1
Total PUFAs, %TE	4.6 ± 0.2	5.3 ± 0.3
TFAs, %TE	0.9 ± 0.1	1.0 ± 0.1
Protein, %TE	17.0 ± 0.5	16.3 ± 0.5
Carbohydrates, %TE	46.7 ± 1.1	46.4 ± 1.0
Alcohol, %TE	2.9 ± 0.5	3.0 ± 0.5
Dietary fiber (AOAC), g/d	20.5 ± 1.0	20.2 ± 1.2
Sodium, g/d	2.6 ± 0.2	2.7 ± 0.2

1 Values are given as untransformed and unadjusted means ± SEMs or *n* (%); *n* = 52 (overall group). Ethnicity was determined by self-report. No significant differences were observed between the baseline characteristics of participants prior to modified and control study visits, as assessed by paired t-tests (continuous variables) and chi-square test (categorical variable). AOAC, Association of Analytical Chemists; HDL-C, HDL cholesterol; LDL-C, LDL cholesterol; TC, total cholesterol; TFA, *trans* fatty acid; %TE, percentage of total energy intake.

2 Assessed by 4-d weighed food diet diaries in the 1-wk run-in period to each study visit.

### Postprandial endothelial function response

The fasting %FMD response was comparable between study days. There was no difference in the postprandial %FMD iAUC or time-course profile (overall treatment) response following consumption of the FA-modified and control sequential test meals (**Table 3**; **Supplemental Figure 2**). However, there was a tendency for an effect of meal dairy FA composition on the AUC for the %FMD response, with a 4% higher response observed after consumption of the FA-modified relative to the control meals (*P* = 0.075).

**TABLE 3:** Fasting and postprandial summary endothelial function and circulating biomarkers of endothelial activation and inflammatory responses following sequential high-fat mixed-meal challenges rich in fatty acid–modified or conventional (control) dairy products in adults with moderate cardiovascular disease risk^1^

	Modified	Control	*P*
%FMD response			
Fasting,^2^ %	4.50 ± 0.27	4.66 ± 0.28	0.112
AUC,^2^ % × min	2040 ± 108	1960 ± 111	0.075
iAUC, % × min	146 ± 78	5 ± 81	0.315
Plasma nitrite			
Fasting, μmol/L	0.14 ± 0.02	0.16 ± 0.02	0.570
AUC, μmol/L × min	64.6 ± 7.9	65.5 ± 8.5	0.980
iAUC, μmol/L × min	4.3 ± 4.9	-0.8 ± 4.7	0.480
Plasma nitrate			
Fasting,^2^ μmol/L	16.1 ± 0.9	16.6 ± 1.0	0.970
AUC,^2^ μmol/L × min	5280 ± 285	5290 ± 271	0.990
iAUC, μmol/L × min	−1480 ± 249	−1700 ± 248	0.730
Adhesion molecules			
Plasma sVCAM-1			
Fasting,^2^ ng/mL	540 ± 32	554 ± 31	0.623
AUC,^2^ ng/mL × min	226 ± 12	237 ± 13	0.180
iAUC, ng/mL × min	-0.8 ± 3.4	3.7 ± 4.5	0.489
Plasma sICAM-1			
Fasting, ng/mL	88.2 ± 6.9	84.3 ± 7.3	0.534
AUC, ng/mL × min	37.8 ± 3.0	36.4 ± 3.1	0.461
iAUC, ng/mL × min	0.7 ± 1.2	1.0 ± 1.5	0.959
Plasma E-selectin			
Fasting, ng/mL	25.2 ± 2.0	25.2 ± 1.7	0.953
AUC, ng/mL × min	10.3 ± 0.8	10.3 ± 0.7	0.992
iAUC, ng/mL × min	−0.28 ± 0.2	−0.27 ± 0.1	0.934
Plasma P-selectin			
Fasting,^2^ ng/mL	25.7 ± 1.4	26.7 ± 1.5	0.257
AUC,^2^ ng/mL × min	10.7 ± 0.6	10.7 ± 0.6	0.912
iAUC, ng/mL × min	−0.05 ± 0.2	−0.51 ± 0.2	0.090
Whole blood culture LPS-stimulated cytokines (per 10^3^ monocytes)			
TNF-α			
Fasting,^2^ μg	12.3 ± 0.9	11.5 ± 0.6	0.729
AUC,^2^ mg × min	5.00 ± 0.3	4.99 ± 0.3	0.250
iAUC, mg × min	−0.18 ± 0.2	0.16 ± 0.2	0.319
IL-6			
Fasting,^2^ μg	82.6 ± 4.3	79.1 ± 4.2	0.869
AUC,^2^ mg × min	32.8 ± 1.7	33.2 ± 1.7	0.453
iAUC, mg × min	−1.88 ± 1.0	0.03 ± 1.2	0.343
IL-1β			
Fasting,^2^ μg	27.4 ± 1.5	26.2 ± 1.3	0.516
AUC,^2^ mg × min	11.9 ± 0.6	12.5 ± 0.6	0.181
iAUC, mg × min	0.36 ± 0.4	1.50 ± 0.4	0.024
IL-8			
Fasting, μg	124 ± 10	130 ± 10	0.653
AUC, mg × min	48.6 ± 4.1	51.3 ± 6.0	0.502
iAUC, mg × min	−3.35 ± 2.4	−3.13 ± 4.4	0.921
IL-10			
Fasting, μg	0.77 ± 0.08	0.91 ± 0.09	0.060
AUC,^2^ mg × min	0.28 ± 0.02	0.35 ± 0.04	0.083
iAUC, mg × min	−0.04 ± 0.02	−0.03 ± 0.02	0.645

1 Values are untransformed and unadjusted means ± SEMs. For all variables, *n* = 50, except for nitrite and nitrate, *n* = 49; TNF-α, IL-1β, IL-6, IL-10, *n* = 48; %FMD response, *n* = 45; and IL-8, *n* = 43. The time interval for AUC and iAUC: 420 min for all variables. Linear mixed-model analyses were used to calculate overall treatment effect in postprandial summary measures, with adjustments made for fixed effects of period, treatment, gender, age, and BMI. Participant was included as a random effPostprandial endothelial function response intercellular adhesion molecule 1; sVCAM-1, soluble vascular adhesion molecule 1, %FMD, percentage of flow-mediated dilatation.

2 Indicates data were log-transformed prior to analysis.

### Postprandial lipid, glucose, and insulin responses

Fasting serum concentrations of TGs, apoB, NEFAs, glucose, and insulin were comparable between study days. There was no differential impact of meal dairy FA composition on the postprandial lipid (TGs and NEFAs), apoB, glucose, and insulin summary measures (**Table 4**) or time-course responses (0–480 min) (data not shown).

**TABLE 4:** Fasting and postprandial serum lipids, glucose, and insulin responses following sequential high-fat mixed-meal challenges rich in fatty acid–modified or conventional (control) dairy products in adults with moderate cardiovascular disease risk^1^

	Modified	Control	*P*
TGs^2^			
Fasting, mmol/L	1.25 ± 0.08	1.15 ± 0.06	0.163
Cmax, mmol/L	2.83 ± 0.15	2.58 ± 0.12	0.080
Tmax, min	355 ± 9	334 ± 9	0.045
AUC, mmol/L × min	950 ± 53	879 ± 41	0.163
iAUC, mmol/L × min	351 ± 26	325 ± 23	0.651
apoB			
Fasting,^2^ mg/mL	1.01 ± 0.03	1.00 ± 0.03	0.324
Cmax,^2^ mg/mL	1.03 ± 0.03	1.04 ± 0.03	0.725
Tmax, min	212 ± 24	207 ± 22	0.790
AUC,^2^ mg/mL × min	472 ± 14	470 ± 14	0.477
iAUC, mg/mL × min	-14 ± 3	-11 ± 2	0.284
NEFAs			
Fasting,^2^ μmol/L	557 ± 25	554 ± 31	0.412
Cmin_30–330_,^2^ μmol/L	113 ± 5	111 ± 6	0.212
Tmin_30–330_,^2^ min	142 ± 6	135 ± 7	0.394
Suppression_30–330_,^2^ %	79 ± 1	77 ± 2	0.318
Cmax_120–480_,^2^ μmol/L	436 ± 19	481 ± 21	0.083
Tmax_120–480_,^2^ min	361 ± 5	360 ± 6	0.806
AUC_120–480_,^2^ mmol/L × min	91 ± 3	97 ± 4	0.192
iAUC_120–480_, mmol/L × min	45 ± 3	51 ± 4	0.223
Glucose			
Fasting,^2^ mmol/L	5.41 ± 0.11	5.39 ± 0.10	0.870
Cmax,^2^ mmol/L	8.27 ± 0.22	7.99 ± 0.21	0.086
Tmax, min	230 ± 25	243 ± 25	0.620
AUC,^2^ mmol/L × min	2940 ± 64	2870 ± 64	0.055
iAUC,^2^ mmol/L × min	347 ± 41	286 ± 40	0.145
Insulin			
Fasting, pmol/L	41.2 ± 3.6	41.3 ± 4.0	0.948
Cmax, pmol/L	551 ± 51	509 ± 42	0.108
Tmax, min	170 ± 25	222 ± 25	0.062
AUC, μmol/L × min	121 ± 11	114 ± 9	0.204
iAUC, μmol/L × min	102 ± 9	95 ± 8	0.151

1 Values are untransformed and unadjusted means ± SEMs. For all variables, *n* = 46, except *n* = 49 for apoB and *n* = 43 for NEFAs. Time interval for AUC and iAUC: 480 min for all variables, except for 360 min NEFA. Linear mixed-model analyses were used to calculate overall treatment effect in postprandial summary measures, with adjustments made for fixed effects of period, treatment, gender, age, and BMI. Participant was included as a random effect. For all outcome measures, *P* ≤ 0.01 was deemed significant to acknowledge multiplicity. Cmax, maximum concentration; Cmin, minimum concentration; iAUC, incremental AUC; NEFA, nonesterified fatty acid; TG; triacylglycerol; Tmax, time to reach maximum concentration; Tmin, time to reach minimum concentration.

2 Indicates data were log-transformed prior to analysis.

As outlined in **Supplemental Table 1**, subanalyses of AUC and iAUC responses for the time period between the breakfast and lunch meals (0–330 min) and after the lunch meal (330–480 min) revealed differences in the TG and glucose responses between the modified and control dairy fat meals. For the TG response, there was a 2.8% higher iAUC after the modified dairy compared with the control breakfast meal (0–330 min) (*P* = 0.009). No other pre- or post-lunch differences in AUC or iAUC were evident between the modified and control dairy meals (i.e., *P* > 0.01).

### Postprandial response for circulating markers of endothelial activation and inflammation

Baseline (fasting) concentrations of all plasma markers of endothelial activation and inflammation were comparable between study days. Meal dairy FA composition had no effect on postprandial endothelial activation or whole blood culture LPS-stimulated cytokine summary measures (Table 3) or time-course profile responses (data not shown), with the exception of a tendency towards a 76% lower iAUC for the LPS-stimulated whole-blood IL-1β response after consumption of the modified, relative to the control, dairy fat meals (*P* = 0.024).

### Postprandial plasma total lipid FA responses

**FIGURE 2:**
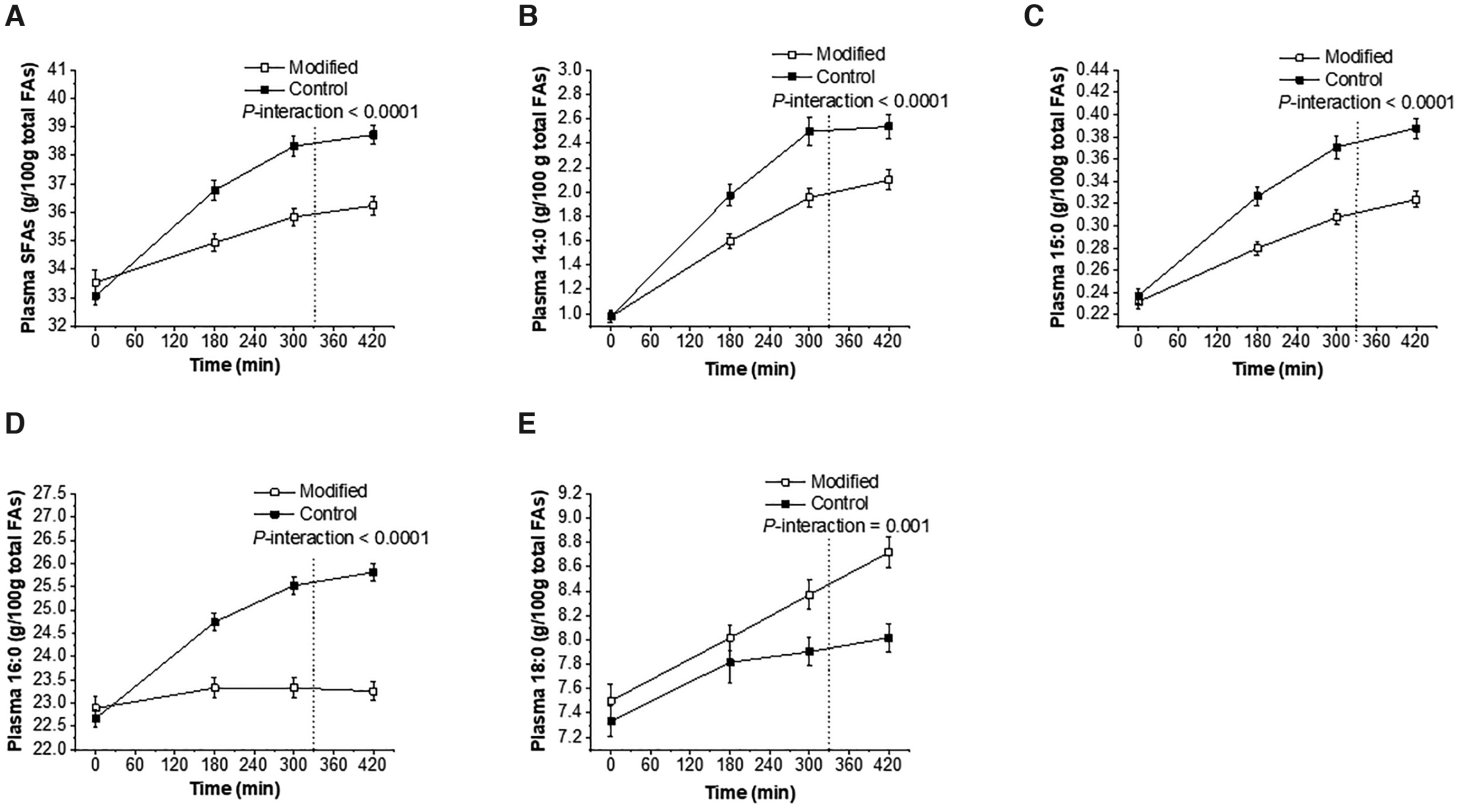
Time-course profiles of postprandial plasma total lipid FAs in response to sequential high-fat mixed-meal challenges (breakfast at 0 min and lunch at 330 min) rich in FA-modified or conventional dairy products (control) in adults at moderate cardiovascular risk for total SFAs (A), 14:0 (B), 15:0 (C), 16:0 (D), and 18:0 (E). Values are untransformed and unadjusted means ± SEMs, *n* = 47–48. The dotted lines represent the timing of the second meal (330 min). Linear mixed-model analysis was used to explore the effects of treatment and time, with an adjustment made in all cases for fixed effects of period, time, treatment, gender, age, and BMI. Participant was included as a random effect. *P* ≤ 0.01 was deemed significant to acknowledge multiplicity. FA, fatty acid.

As outlined in **Table 5**, baseline (fasting) abundance of all plasma total lipid FAs was comparable between treatments. The abundance of total SFAs, 14:0 (myristic acid), 15:0 (pentadecanoic acid), and 16:0 (palmitic acid) was significantly lower following the FA-modified dairy compared with the control dairy fat meals (all *P* < 0.0001 for the AUC, iAUC, and the time-course interaction effect) (Table 5; **Figure 2**). The abundance of 18:0 (stearic acid) over the postprandial period was higher following the FA-modified compared with the control dairy meals (*P* = 0.0002 and *P* = 0.001 for the AUC response and the time-course interaction effect, respectively).

**TABLE 5:** Fasting and postprandial summary responses of selected plasma total lipid fatty acids following sequential high-fat mixed-meal challenges rich in fatty acid–modified or conventional (control) dairy products in adults with moderate cardiovascular disease risk^1^

	Modified	Control	*P*
SFAs			
12:0			
Fasting, g/100 g total FAs	0.09 ± 0.01	0.09 ± 0.01	0.489
AUC,^2^ g/100 g total FAs × min	47 ± 7	68 ± 13	0.446
iAUC,^2^ g/100 g total FAs × min	7 ± 7	29 ± 14	0.446
14:0^2^			
Fasting, g/100 g total FAs	0.98 ± 0.05	0.98 ± 0.05	0.861
AUC, g/100 g total FAs × min	689 ± 24	835 ± 28	<0.0001
iAUC, g/100 g total FAs × min	279 ± 15	424 ± 25	<0.0001
15:0			
Fasting, g/100 g total FAs	0.23 ± 0.01	0.24 ± 0.01	0.468
AUC, g/100 g total FAs × min	119 ± 2	138 ± 3	<0.0001
iAUC,^2^ g/100 g total FAs × min	22 ± 1	39 ± 2	<0.0001
16:0			
Fasting,^2^ g/100 g total FAs	22.89 ± 0.25	22.67 ± 0.19	0.451
AUC,^2^ g/100 g total FAs × min	9760 ± 91	10,400 ± 73	<0.0001
iAUC, g/100 g total FAs × min	142 ± 33	837 ± 48	<0.0001
17:0			
Fasting, g/100g total FAs	0.28 ± 0.01	0.28 ± 0.01	0.537
AUC,^2^ g/100 g total FAs x min	117 ± 3	128 ± 3	0.603
iAUC,^2^ g/100 g total FAs × min	1 ± 3	12 ± 4	0.458
18:0			
Fasting,^2^ g/100 g total FAs	7.50 ± 0.13	7.33 ± 0.12	0.103
AUC,^2^ g/100 g total FAs × min	3410 ± 42	3260 ± 45	0.0002
iAUC, g/100 g total FAs × min	255 ± 38	181 ± 43	0.132
Total SFAs^3^			
Fasting,^2^ g/100 g total FAs	33.30 ± 0.36	32.91 ± 0.28	0.277
AUC,^2^ g/100 g total FAs × min	14,700 ± 126	15,400 ± 111	<0.0001
iAUC, g/100g total FAs × min	741 ± 69	1580 ± 109	<0.0001
MUFAs			
*cis*-9 18:1			
Fasting,^2^ g/100 g total FAs	21.75 ± 0.38	21.56 ± 0.35	0.923
AUC,^2^ g/100 g total FAs × min	9350 ± 161	8730 ± 146	<0.0001
iAUC, g/100 g total FAs × min	219 ± 76	-323 ± 67	<0.0001
Total *cis-*18:1^4^			
Fasting,^2^ g/100 g total FAs	23.53 ± 0.32	23.36 ± 0.30	0.975
AUC,^2^ g/100 g total FAs × min	10,100 ± 133	9460 ± 124	<0.0001
iAUC, g/100 g total FAs × min	204 ± 63	−353 ± 56	<0.0001
Total *cis*-MUFAs^5^			
Fasting,^2^ g/100 g total FAs	25.19 ± 0.36	24.99 ± 0.32	0.996
AUC,^2^ g/100 g total FAs × min	10,800 ± 150	10,300 ± 139	<0.0001
iAUC,^2^ g/100 g total FAs × min	272 ± 67	−238 ± 59	<0.0001
*trans*-9 18:1			
Fasting, g/100 g total FAs	0.12 ± 0.01	0.12 ± 0.01	0.309
AUC, g/100 g total FAs × min	85 ± 3	53 ± 2	<0.0001
iAUC,^2^ g/100 g total FAs × min	33 ± 5	4 ± 4	<0.0001
*trans*-10 18:1			
Fasting,^2^ g/100 g total FAs	0.09 ± 0.01	0.10 ± 0.01	0.239
AUC, g/100 g total FAs × min	222 ± 9	57 ± 2	<0.0001
iAUC, g/100 g total FAs × min	184 ± 9	16 ± 3	<0.0001
*trans*-11 18:1			
Fasting,^2^ g/100 g total FAs	0.14 ± 0.01	0.15 ± 0.01	0.421
AUC,^2^ g/100 g total FAs × min	100 ± 3	102 ± 2	0.198
iAUC, g/100 g total FAs × min	41 ± 3	41 ± 3	0.811
Total *trans-*18:1^6^			
Fasting,^2^ g/100 g total FAs	0.56 ± 0.03	0.57 ± 0.03	0.679
AUC,^2^ g/100 g total FAs × min	657 ± 19	358 ± 7	<0.0001
iAUC, g/100 g total FAs × min	422 ± 22	118 ± 12	<0.0001
Total *trans*-MUFAs^7^			
Fasting,^2^ g/100 g total FAs	1.03 ± 0.03	1.02 ± 0.03	0.934
AUC,^2^ g/100 g total FAs × min	855 ± 21	552 ± 9	<0.0001
iAUC, g/100 g total FAs × min	424 ± 22	122 ± 13	<0.0001
Total TFAs^8^			
Fasting, g/100 g total FAs	1.36 ± 0.04	1.36 ± 0.04	0.699
AUC, g/100 g total FAs × min	1000 ± 24	695 ± 11	<0.0001
iAUC, g/100 g total FAs × min	434 ± 25	124 ± 16	<0.0001
PUFAs			
Total CLAs^9^			
Fasting,^2^ g/100 g total FAs	0.19 ± 0.01	0.20 ± 0.01	0.561
AUC,^2^ g/100 g total FAs × min	118 ± 4	108 ± 4	0.019
iAUC,^2^ g/100g total FAs × min	39 ± 3	25 ± 2	0.001
Total n–3 PUFAs^10^			
Fasting,^2^ g/100 g total FAs	3.72 ± 0.12	3.86 ± 0.15	0.426
AUC,^2^ g/100 g total FAs × min	1460 ± 50	1480 ± 52	0.786
iAUC,^2^ g/100 g total FAs × min	−106 ± 24	−143 ± 29	0.211
Total n–6 PUFAs^11^			
Fasting, g/100 g total FAs	34.94 ± 0.66	35.43 ± 0.59	0.544
AUC,^2^ g/100 g total FAs × min	13,400 ± 241	13,600 ± 217	0.440
iAUC,^2^ g/100 g total FAs × min	-1250 ± 138	-1250 ± 139	0.896

1 Values are untransformed and unadjusted means ± SEMs. For all variables, *n* = 47–48. The time interval for AUC and iAUC: 420 min for all variables. Linear mixed-model analyses were used to calculate overall treatment effect in postprandial summary measures, with adjustments made for fixed effects of period, treatment, gender, age, and BMI. Participant was included as a random effect. For all outcome measures, *P* ≤ 0.01 was deemed significant to acknowledge multiplicity. CLA, conjugated linoleic acid; FA, fatty acid; iAUC, incremental AUC; TFA, *trans* fatty acid.

2 Indicates data were log-transformed prior to analysis.

3 Total SFAs include: 6:0, 7:0, 8:0, 9:0, 10:0, 11:0, 12:0, 13:0 iso, 13:0 anteiso, 13:0, 14:0 iso, 14:0, 15:0 anteiso, 15:0, 16:0 iso, 16:0, 17:0 iso, 17:0 anteiso, 17:0, 18:0 iso, 18:0, 19:0, 20:0, 22:0, and 24:0.

4 Total *cis*-18:1 include: *cis*-9 18:1, *cis*-11 18:1, *cis*-12 18:1, *cis*-13 18:1, *cis*-14 18:1, *cis*-15 18:1, and *cis*-16 18:1.

5 Total *cis*-MUFAs include: *cis*-9 10:1, *cis*-10 11:1, *cis*-9 12:1, 13:1 (unknown bond position), *cis*-9 14:1, *cis*-10 15:1, *cis*-9 16:1, *cis*-13 16:1, *cis*-10 17:1, *cis*-9 17:1, *cis*-9 18:1, *cis*-11 18:1, *cis*-12 18:1, *cis*-13 18:1, *cis*-14 18:1 *cis*-15 18:1, *cis*-16 18:1, 19:1 (unknown bond position), *cis*-5 20:1, *cis*-8 20:1, *cis*-11 20:1, *cis*-13 22:1, and *cis*-15 24:1.

6 Total *trans* 18:1 include: *trans*-4 18:1, *trans*-6 18:1, *trans*-7 18:1, *trans*-8 18:1, *trans*-9 18:1, *trans*-10 18:1, *trans*-11 18:1, *trans*-12 18:1, *trans*-15 18:1, and *trans*-16 18:1.

7 Total *trans*-MUFAs include: *trans*-9 14:1, *trans*-9 16:1, *trans*-11 16:1, *trans*-13 16:1, *trans*-10 17:1, *trans*-4 18:1, *trans*-6 18:1, *trans*-7 18:1, *trans*-8 18:1, *trans*-9 18:1, *trans*-10 18:1, *trans*-11 18:1, *trans*-12 18:1, *trans*-15 18:1, and *trans*-16 18:1.

8 Total TFAs include: *trans*-18:1, *trans*-11, 15 18:2, *trans*-9, 12 18:2, *cis*-9, *trans*-13 18:2, *cis*-10, *trans*-14 18:2, *cis*-9, *trans*-12 18:2, *trans*-9, 12 18:2, *trans*-11, *cis*-15 18:2, and *trans*-12, *cis*-15 18:2.

9 Total CLAs include a peak, which contains mainly *cis*-9, *trans*-11 CLA, but also *trans*-7, *cis*-9 CLA, *trans*-8, *cis*-10 CLA, and *trans*-6, *cis*-8 CLA.

10 Total n–3 PUFAs include: *trans*-11, 15 18:2, *trans*-11, *cis*-15 18:2, *trans*-12, *cis*-15 18:2, *cis*-9, 12, 15, 18:3, *cis*-11, 14, 17 20:3, *cis*-5, 8, 11, 14, 17 20:5, *cis*-7, 10, 13, 16, 19 22:5, and *cis*-4, 7, 10, 13, 16, 19 22:6.

11 Total n–6 PUFAs include: *trans*-9, 12 18:2, *cis-9, trans*-12 18:2, *trans*-9, *cis*-12 18:2, *cis*-9, 12 18:2, *cis*-6, 9, 12 18:3, *cis*-11, 14 20:2, *cis*-8, 11, 14 20:3, *cis*-5, 8, 11, 14 20:4, *cis*-13, 16 20:2, and *cis*-7, 10, 13, 16 22:4.

**FIGURE 3:**
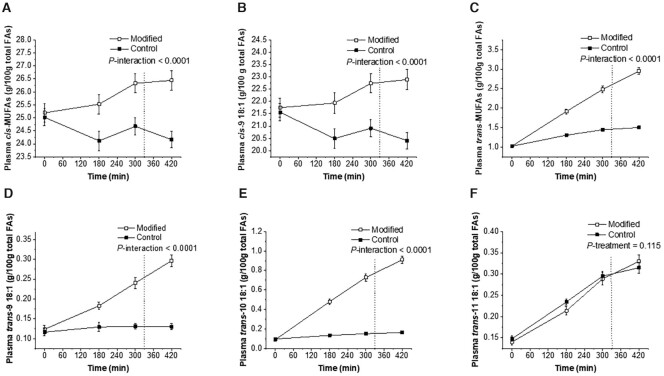
Time-course profiles of postprandial plasma total lipid FAs in response to sequential high-fat mixed-meal challenges (breakfast at 0 min and lunch at 330 min) rich in FA-modified or conventional dairy products (control) in adults at moderate cardiovascular risk for *cis*-MUFAs (A), *cis*-9 18:1 (B), *trans*-MUFAs (C), *trans*-9 18:1 (D), *trans*-10 18:1 (E), and *trans*-11 18:1 (F). Values are untransformed and unadjusted means ± SEMs, *n* = 49. The dotted lines represent the timing of the second meal (330 min). Linear mixed-model analysis was used to explore the effects of treatment and time, with an adjustment made in all cases for fixed effects of period, time, treatment, gender, age, and BMI. Participant was included as a random effect. *P* ≤ 0.01 was deemed significant to acknowledge multiplicity. FA, fatty acid.

The abundance of total *cis*-MUFAs (predominantly *cis*-9 18:1 (oleic acid)), total *trans*-18:1 (including *trans*-9 18:1 (elaidic acid) and *trans*-10 18:1 (octadecenoic acid)), and total *trans*-MUFAs increased in the plasma total lipid FA pool following the consumption of the FA-modified dairy fat meals compared with control (*P* < 0.0001 for the AUC, iAUC, and the time-course interaction effect) (Table 5; **Figure 3**). The increase in the proportion of *trans*-11 18:1 (vaccenic acid) was similar between control and FA-modified dairy meals (AUC, iAUC, and time-course overall treatment effect: *P* > 0.05). The abundance of conjugated linoleic acids also increased following consumption of the modified, relative to the control, dairy products (iAUC: *P* = 0.001; and the time-course overall treatment effect: *P* = 0.006).

## Discussion

This study highlights that, in adults at moderate CVD risk, acute consumption of high-fat mixed meals rich in modified (SFA-reduced, MUFA-rich) dairy products did not differentially affect postprandial endothelial function or systemic biomarkers of cardiometabolic health, relative to meals that contained control (conventional) dairy products. Postprandial abundance of total lipid FAs in the plasma following sequential FA-modified meal consumption largely reflected the partial replacement of SFAs with MUFAs in our dairy-rich test meals.

Endothelial dysfunction in the postprandial state has been attributed to a progressive detrimental effect on CVD risk (12, 13). Here we demonstrate that acute consumption of dairy meals varying in FA composition did not lead to different postprandial endothelial function responses, although there was a tendency for a beneficial increase in the AUC (but not iAUC) for the %FMD response following consumption of the modified dairy meals. This observation largely agrees with previous acute investigations, which found that consumption of high-fat mixed meals containing specific dietary fats (SFAs, MUFAs, and n–6 PUFAs) did not differentially influence impairments observed in postprandial endothelial function, as assessed by FMD (35–37). In contrast, the parallel LIPGENE postprandial study by Perez-Martinez et al. (38) found that patients with the metabolic syndrome assigned to an MUFA-rich test breakfast (65% total fat: 12% SFAs, 43% MUFAs) had a significantly higher postprandial FMD response compared with those assigned to other test meal conditions, including a fat-matched SFA-rich breakfast (38% SFAs, 21% MUFAs) (38). This study also highlighted that the availability of postprandial NO synthase was greater and plasma sICAM-1 responses were lower following the ingestion of a high-MUFA, relative to the SFA-rich, meal, which contained predominantly butter (38). Rundblad and colleagues (39) demonstrated that a high-fat meal containing butter, but not fermented dairy products (such as cheese), increased postprandial sICAM-1 and sVCAM-1 concentrations. As our modified and control test meals contained a combination of dairy products varying in food matrix and fermentation, this may partly explain why we did not have differential postprandial endothelial activation in response to our high-fat dairy products. However, it is difficult to draw meaningful comparisons between our study and the findings of Perez-Martinez et al. (38), as the postprandial test meal in the latter study was administered at the end of a 12-wk period of dietary FA manipulation. However, another plausible explanation for contrasting endothelial function outcomes could be that our FA-modified and control sequential test meals only had relatively modest difference (∼12 g) in both the content of total SFAs and *cis-*MUFAs, compared with the LIPGENE postprandial study where test-meal FA modification was not limited specifically to dairy products (38).

Elevated systemic inflammation in the postprandial period is linked to increased risk for atherosclerosis and insulin resistance, with a single high-fat meal known to increase proinflammatory cytokine concentrations, particularly IL-6 (40). Our study findings suggested that the FA composition of the dairy products affected postprandial systemic inflammatory responses to a similar extent, apart from a tendency for a potentially favorable reduction in the whole blood culture LPS-stimulated IL-1β iAUC response following modified dairy meals. This finding may be related to higher MUFA content of these dairy products since a study conducted in insulin-resistant patients found the fasting plasma FA concentration of oleic acid (*cis*-9 18:1) to be negatively associated with systemic IL-1β concentrations (41). Although not directly comparable, our study agrees with a previous observation highlighting that the dairy content of high-fat meals did not differently affect postprandial IL-6 and TNF-α responses in healthy men (42). Similarly, the FA quality of high-fat meals did not elicit differences in postprandial IL-6 concentrations in metabolic syndrome patients (43) or obese and lean women (44).

We observed no differential effect of our sequential dairy test meal challenge on postprandial lipid, glucose, or insulin concentrations. This finding is largely in agreement with previous acute studies that compared the metabolic effects of sequential high-fat meals rich in SFAs, MUFAs, and n–6 PUFAs among postmenopausal women (35) and single high-fat meals (40 g total fat) containing different proportions of MUFAs (12%, 17%, and 24% of energy) in healthy men (45). In subanalyses, we found a higher TG iAUC response following the modified breakfast meal (0–330 min), relative to the control meal. In line with this, Tholstrup et al. (46) observed higher postprandial plasma and chylomicron TG responses to a high-fat meal containing FA-modified butter at 4 h postprandially in healthy young men, when compared with a conventional Danish butter. However, postprandial responses were assessed after a 4-wk isoenergetic dietary intervention (with 16% of 12:0–16:0 replaced mainly with *cis*-9 18:1 and 18:0 in the FA-modified butter diet) (46).

Supplementation of the dairy-cow diet with unprotected HOS oil by our group led to a proportion of milk SFAs being replaced by *cis*-MUFAs, as well as *trans*-MUFAs through the process of biohydrogenation of unsaturated FAs by rumen bacteria (16). Consequently, our FA-modified, 2-meal challenge each contained a 3-g (2-fold) higher *trans*-MUFA content compared with the conventional dairy meals. Our bovine supplementation strategy also led to alterations in the *trans*-18:1 isomer profile, with a shift from the formation of predominantly *trans*-11 in conventional milk fat toward a greater proportion of *trans*-10 (and to a lesser extent, *trans*-9) intermediates in modified milk fat, which was primarily as a result of isomerization of *cis*-9 18:1 within the rumen (16). The postprandial composition of the plasma total lipid FA pool largely resembled that of the ingested dairy fats (16). We observed a reduction in total SFAs (including 14:0, 15:0, and 16:0) and an increase in the abundance of 18:0, total *cis*-MUFAs (primarily *cis*-9 18:1), and total *trans-*MUFAs (particularly *trans*-9 and *trans*-10 18:1 isomers) in the plasma total lipid pool following consumption of the sequential modified dairy, relative to the control, meals. It may be considered that the higher total *trans*-18:1 content of our FA-modified dairy meals, and abundance of *trans*-18:1 isomers in the plasma lipid pool, might have counteracted the impairment of endothelial function and inflammation in response to the sequential high-fat meals linked to favorable postprandial changes in the abundance of plasma total SFAs and *cis*-MUFAs following consumption of the FA-modified dairy meals. Indeed, increased intake of *trans*-9 18:1 (elaidic acid), one of the major *trans* isomers produced industrially by the partial hydrogenation of vegetable oils, is adversely linked to cardiovascular health outcomes (47, 48). Cross-sectional data generated from the Nurses’ Health Study illustrated that intake of *trans*-9 18:1 was positively associated with E-selectin, sVCAM-1, sICAM-1, and soluble TNF-α receptor 2 in women (49). In support of differences in biological mechanisms between *trans-*18:1 isomers, Iwata et al. (50) reported that a 180-min treatment of human endothelial cells with increasing concentrations (≤0.1 mM) of *trans-*9 18:1 was associated with increased NF-κB activation and impaired NO production, whereas *trans*-vaccenic acid (*trans*-11 18:1) was not related to either of these responses. Taking into consideration that the concentrations of total plasma *trans* FAs in humans have been shown to be as high as 0.6 mM in the fasted state (51), the FA concentrations used in the aforementioned in vitro study could be deemed physiological (52).

Strengths of the current study include the double-blinded, randomized design and use of a sequential high-fat mixed-meal challenge, which provided a more accurate reflection of Western dietary patterns (32). We studied adults at moderate CVD risk but acknowledge that our findings may not be generalizable across different groups, including young, healthy populations. Our modified foods largely reflected the natural FA profile of milk produced by an HOS-oil bovine feeding strategy (16) but our strategy increased the concentration of *trans-*18:1 isomers in milk, which were incorporated into the plasma total lipid pool following acute consumption. Further research is warranted to examine the cardiometabolic health effects of FA-reformulated dairy foods that are produced following rumen-protected bovine supplementation strategies, and thus contain a reduced formation of *trans*-18:1 intermediates.

In conclusion, consumption of sequential high-fat meals rich in FA-modified dairy products led to a lower abundance of total SFAs (including 14:0, 15:0, and 16:0) and higher abundance of 18:0 and total *cis*- and *trans*-MUFAs (predominantly *trans*-18:1 isomers) in the postprandial plasma total lipid pool, when compared with control dairy meals. Meal dairy FA consumption did not elicit significant differences in postprandial endothelial function or systemic cardiometabolic risk markers in our cohort of adults at moderate CVD risk. Previous work has shown that chronic consumption of FA-modified dairy products may have a beneficial impact on the fasting cholesterol profile (17, 53) and endothelial function (17). As the FA composition of the background diet may be of greater health importance than isolated acute differences in FA intake (54), the postprandial effects of long-term FA-modified dairy product consumption need further investigation.

## Supplementary Material

nxab050_Supplemental_File

## Notes

Supported by the UK Medical Research Council (MR/K020218/1). Arla Foods UK and AAKUK supplied in kind the commercially available dairy products and high-oleic sunflower oil, respectively, according to our specification.

Author disclosures: JAL is Deputy Chair of the UK Scientific Advisory Committee for Nutrition (SACN) and was an expert on SACN's Saturated Fats Working Group. All other authors report no conflicts of interest. JAL is a member of the Journal's Editorial Board and played no role in the Journal's evaluation of the manuscript. The funders of the study had no role in study design, data collection, data analysis, data interpretation or writing of the manuscript.

Supplemental Table 1 and Supplemental Figures 1 and 2 are available from the “Supplementary data” link in the online posting of the article and from the same link in the online table of contents at https://academic.oup.com/jn/.

OM and DV contributed equally to this study.

Abbreviations used: CVD, cardiovascular disease; FA, fatty acid; FID, flame ionization detector; FMD, flow-mediated dilatation; HOS, high-oleic sunflower oil; iAUC, incremental AUC; IPAQ, International Physical Activity Questionnaire; NEFA, nonesterified fatty acid; RESET, REplacement of SaturatEd fat in dairy on Total cholesterol; sICAM-1, soluble intercellular adhesion molecule-1; sVCAM-1, soluble vascular cell adhesion molecule-1; TG, triacylglycerol; UHT, ultra-high temperature; %FMD, percentage flow-mediated dilatation.
