# Supplementary material for: Postprandial Fatty Acid Profile, but Not Cardiometabolic Risk Markers, Is Modulated by Dairy Fat Manipulation in Adults with Moderate Cardiovascular Disease Risk: The Randomized Controlled REplacement of SaturatEd fat in dairy on Total cholesterol (RESET) Study
Source: J Nutr. 2021 Mar 23;151(7):1755–68. doi: 10.1093/jn/nxab050 (PMC8327197; doi:10.1093/jn/nxab050)
Supplement: nxab050_Supplemental_File [file nxab050_supplemental_file.docx]

**Postprandial Fatty Acid Profile, but Not Cardiometabolic Risk Markers, Is Modulated**

**by Dairy Fat Manipulation in Adults with Moderate Cardiovascular Disease Risk: The**

**Randomized Controlled REplacement of SaturatEd fat in dairy on Total cholesterol**

**(RESET) Study, Markey et al., Online Supplementary Material**

**Supplemental Table 1** Postprandial summary serum lipids, glucose, and insulin responses in adults with moderate cardiovascular risk before and after lunch meals rich in fatty acid-modified or conventional (control) dairy products ^1^

|  | Modified | Control | *P*^3^ |
| --- | --- | --- | --- |
| TAG  Before lunch |  |  |  |
| AUC^2^, mmol/L x min | 591 ± 33 | 556 ± 26 | 0.255 |
| iAUC^2^, mmol/L x min | 180 ± 14 | 175 ± 13 | 0.009 |
| After lunch |  |  |  |
| AUC^2^, mmol/L x min | 363 ± 22 | 323 ± 17 | 0.057 |
| iAUC^2^, mmol/L x min | 14 ± 8 | 11 ± 9 | 0.467 |
| apoB |  |  |  |
| Before lunch |  |  |  |
| AUC_0-300_, mg/mL x min | 297 ± 9 | 294 ± 9 | 0.124 |
| iAUC_0-300_^2^, mg/mL x min | -7 ± 2 | -6 ± 1 | 0.401 |
| After lunch |  |  |  |
| AUC_360-480_, mmol/L x min | 118 ± 4 | 117 ± 4 | 0.181 |
| iAUC_360-480_^2^, mmol/L x min | -1 ± 1 | 3 ± 4 | 0.251 |
| Glucose |  |  |  |
| Before lunch |  |  |  |
| AUC^2^, mmol/L x min | 1950 ± 43 | 1880 ± 43 | 0.045 |
| iAUC, mmol/L x min | 163 ± 27 | 106 ± 28 | 0.063 |
| After lunch |  |  |  |
| AUC^2^, mmol/L x min | 990 ± 27 | 988 ± 28 | 0.803 |
| iAUC^2^, mmol/L x min | 223 ± 23 | 229 ± 22 | 0.794 |
| Insulin |  |  |  |
| Before lunch |  |  |  |
| AUC^2^, µmol/L x min | 81 ± 7 | 76 ± 6 | 0.241 |
| iAUC^2^, µmol/L x min | 68 ± 6 | 68 ± 5 | 0.168 |
| After lunch |  |  |  |
| AUC^2^, mmol/L x min | 41 ± 4 | 39 ± 3 | 0.934 |
| iAUC^2^, mmol/L x min | 31 ± 4 | 29 ± 3 | 0.772 |

^1^Values are untransformed and unadjusted means ± SEMs. *n* = 46 for all variables, except for *n* = 45 for apoB. AUC and iAUC responses after lunch and *n* = 44 glucose AUC and iAUC responses after lunch. The time interval for AUC and iAUC response before lunch was: 330 min for all variables, except for 0-300 min for apoB. The time interval for AUC and iAUC responses after lunch was: 150 min for all variables, except for 360-480 min for apoB. apoB, apolipoprotein B; AUC, incremental area under the curve; iAUC, incremental AUC; TAG; triacylglycerol.

^2^Indicates data were *log* transformed prior to analysis.

^3^Linear mixed model analyses were used to calculate overall treatment effect in postprandial summary measures, with adjustments made for fixed effects of period, treatment, gender, age, and BMI. Participant was included as a random effect. For all outcome measures, *P* ≤ 0.01 was deemed as significant to acknowledge multiplicity.


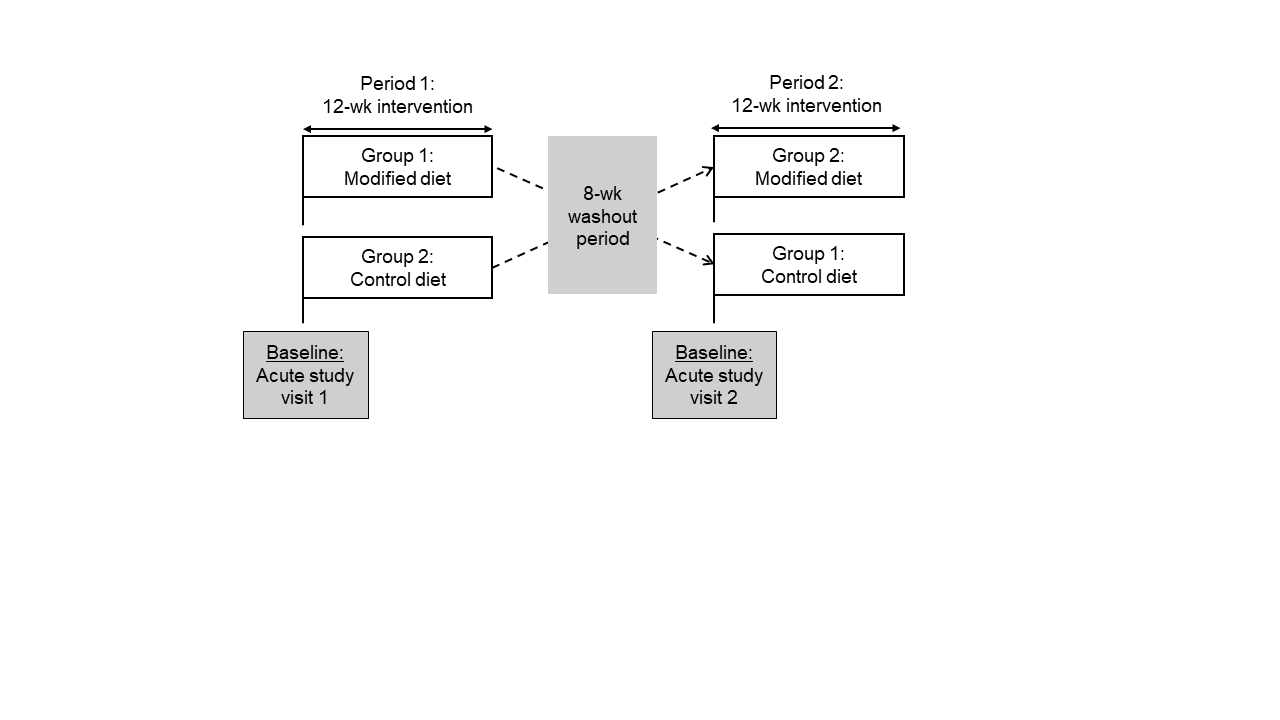


**Supplemental Figure 1** Overview of the acute randomized, double-blind, sequential-meal, crossover dietary trial. Participants were randomly assigned to group 1, starting with a baseline acute study visit where sequential meals rich in fatty acid-modified dairy products were consumed or group 2, starting with a baseline acute study visit where sequential meals rich in control dairy products were consumed prior to a 12-wk dietary intervention period with the same dairy products (period 1). Following an 8-wk washout period, participants crossed over to the alternate dietary intervention period and completed a second baseline acute study visit (period 2).

**Supplemental Figure 2** Time-course profile of postprandial percentage FMD response following sequential high-fat mixed meal challenges (breakfast at 0 min and lunch at 330 min) rich in fatty acid-modified or conventional (control) dairy products in adults at moderate cardiovascular risk. Values are untransformed and unadjusted means ± SEMs, *n* = 45. The dotted line represents the timing of the second meal (330 min). Linear mixed model analysis was used to calculate treatment x time interaction, with an adjustment made for fixed effects of period, time, treatment, gender, age, and BMI. Participant was included as a random effect. *P* < 0.05 was deemed as significant. FMD, flow-mediated dilatation.
